# Editorial: The continuing challenge of medication adherence

**DOI:** 10.3389/fphar.2025.1740788

**Published:** 2025-11-19

**Authors:** John Weinman, Ad A. Kaptein, Rob Horne, John Piette

**Affiliations:** 1 School of Cancer and Pharmaceutical Sciences, King’s College London, London, United Kingdom; 2 Medical Psychology, Leiden University Medical Centre, Leiden, Netherlands; 3 Centre for Behavioural Medicine, University College London, London, United Kingdom; 4 School of Public Health, University of Michigan, Ann Arbor, MI, United States

**Keywords:** medication adherence, key concepts, theory, socio-economic impact, clinical impact, assessment, interventions, future developments

Over 20 years ago the World Health Organisation (WHO) published their influential report, entitled “Adherence to long-term therapies: evidence for action” (Sabaté, 2003), in which they provided an authoritative account of the extent, causes and effects of non-adherence to medication. Although this has been heavily cited and followed by substantial research, treatment adherence continues to be a massive problem with huge impacts on clinical, economic and social outcomes, which the Organisation of Economic Co-Operation and Development (OECD) have described as a major public health scandal (Khan and Socha-Dietrich, 2018).

Since the time of the WHO report, there have been considerable advances in our understanding of the barriers and facilitators of medication adherence. Many interventions have been developed, and these are being increasingly facilitated by the rapid developments in digital technologies and artificial intelligence. While there have been many systematic reviews of the causes of non-adherence and of the interventions for improving adherence, these are widely spread across many journals, which creates a major challenge for the interested clinician or researcher wanting to keep up with the extensive research findings which have accumulated. Moreover, since publication of the WHO report, there is little evidence that adherence has improved and there is continuing evidence that healthcare systems have failed to address the adherence challenge both at a policy and educational level. The purpose of this Research Topic is to allow the reader to gain an overview not only of the state of the art in adherence research and practice but also of some of the continuing issues in this area. Recent science in this area is presented addressing five key interrelated questions:

- What is the nature and prevalence of non-adherence?
- What are the causes of non-adherence?
- What is the best way to assess adherence?
- What are the clinical, social and economic impacts of poor adherence?
- What are the most effective adherence support interventions?

The first paper by Chapman and Chan addresses the first three of these questions. It begins by examining the evolution in the definitions of non-adherence, showing that adherence is now best understood as involving different stages, from treatment initiation through to long-term persistence. They indicate how the estimated prevalence of non-adherence varies according to the stage and type of behaviour since approximately 20% of patients may never start a newly prescribed treatment whereas around double that number fail to take their medicines regularly and even more do not persist over longer periods. They emphasize that part of the variation in these rates depends on how adherence is measured, and they summarise some of the main methods for this. In examining the many causes of non-adherence, they highlight the use of various explanatory models, such as the Capability Opportunity Motivation (COM-B) framework (Jackson et al., 2014) or the Perceptions and Practicalities Approach (Horne et al., 2019).

Even though adherence prevalence estimates vary across studies, there is very consistent evidence that non-adherence has profound effects on a range of important patient and societal outcomes. These impacts are documented in the paper by Achterbosch et al. Their overview of the cumulative findings from 43 systematic reviews provides an extensive picture of the reduced treatment effects, the increased healthcare utilization, morbidity and mortality together with all the financial implications for individuals and healthcare systems. These findings provide a compelling argument for the need to develop effective adherence support interventions in order to reduce the massive clinical, personal and economic costs of non-adherence.

The search for more valid and reliable measures of adherence remains a challenge. Although measures based on electronic monitoring (EM) are often cited as the gold standard (El Alili et al., 2016), these devices are not without their problems, and the next two papers address some of these. Rohay and Dunbar-Jacob examine various operational definitions derived from EM adherence measures and provide important guidelines on calculation methods. However, even the most sophisticated EM methods can only indicate when medicine containers are opened and still do not provide definitive evidence that medicines have been ingested. The search for adherence biomarkers has a chequered history but good evidence is now emerging for the use of chemical adherence testing (CAT). Thus, in the following paper, Rabbitt et al. review the growing number of recent studies showing how CAT is being used in the investigation and management of adherence to antihypertensive medications. Their review indicates that there is a need for greater consistency in the ways in which CAT is used for monitoring and defining adherence. Although CAT could be used to provide patient feedback for improving adherence, this still needs to be developed in an ethical and patient-centric way.

The next two papers provide an overview of the nature and scope of interventions provided by healthcare professionals (HCPs), and those delivered via digital technology. HCP interventions have traditionally targeted patients’ knowledge, understanding and memory since many early studies were based on the assumption that non-adherence was due to a failure of one or more of these processes. However, recent work has shown that reminder-based interventions, although widely used, may have limited impact (Choudhry et al., 2017). The substantial evidence that the causes of non-adherence are many and varied means that interventions need to be carefully chosen to target each individual’s barriers in a personalised way (Allemann et al., 2016). This critical issue is discussed by Crawshaw and McCleary in their overview of HCP led interventions. They show how the variation in the efficacy of these interventions reflects their content and approach. The more effective interventions go beyond the simple provision of information and reminders and are more likely to be tailored to the individual. They also recognise the potentially important role of healthcare systems in embracing the adherence challenge and allowing time and resources for clinicians to engage in adherence support in a meaningful way.

Since clinicians may lack the tools they need for managing the adherence problems they face, the emergence of digital approaches is now seen as a viable way of achieving more widespread interventions, which have been made possible by the global adoption of mobile phone technology. In their paper, Moon and Walsh review the rapid progress in the use of digital adherence interventions. While they outline and recognise the huge potential of digital interventions, they also acknowledge the many challenges inherent in optimising their effective use in practice. There are now a huge number of adherence apps, a large proportion of which are based on providing reminders with varying levels of sophistication and personalisation. Even with the inclusion of artificial intelligence (AI) methodologies, there is still some way to go before their full potential can be realised. There is an enthusiastic but still rather naïve belief that developments in AI, interactive digital technology and precision medicine will solve the adherence problem and, in doing so, obviate the need for HCPs to directly address the adherence challenge. Ultimately these developments may well provide important ways of ensuring that medicines are taken more systematically and effectively but current evidence indicates that they are not instant solutions. For example, a recent review of the use of AI tools in adherence interventions concluded that the evidence is still both limited and weak (Reis et al., 2025). Digital systems will need to be based on a more complete understanding of the individual drivers of non-adherence combined with the targeted use of evidence-based behaviour change techniques (BCTs) and should address patients’ perceptions of the treatment as well as the practicalities of adhering to it (Chapman et al., 2020). A recent example of the types of challenges which app developers need to more effectively tackle has been provided by Wright et al. (2025). Their detailed analysis of the adherence barriers and linked BCTs provides recommendations for the design of apps for supporting better adherence to reliever medication in people with asthma.

The final two papers make use of detailed investigation of experts’ views to identify their perspectives on the adherence challenge and how to improve healthcare practice. The paper by Tan et al. explores the experiences of a group of international clinicians from a range of specialities. Despite the diversity in the countries and specialties represented, all the clinicians acknowledge the central importance of good medication adherence in effective clinical care as well as the difficulties in monitoring and supporting better medicines use. The paper offers a unique perspective by focusing on healthcare professionals’ first-hand experiences with medication non-adherence, a dimension often underrepresented in the literature. Their insights and experiences mirror many of the themes and issues in other papers in this Research Topic. While it is crucial to understand an individual’s reasons for their reluctance or unwillingness to take their medicines in order to provide targeted support, the key role of the HCP has not been sufficiently emphasized. One unfortunate finding from a recent study of HCP’s views of non-adherence was that they perceived that the largest barrier to medication adherence management was lack of patient awareness rather than any shortcoming in their own practice such as the ability to ask about adherence as part of their routine consultations (Hafez et al., 2024). However, almost all the respondents in that survey did recognise their own limitations and the need for better training on medication adherence management. Part of this is due to rushed and poor communication combined with a lack of understanding and skill in the use of behavioural diagnosis and behaviour change techniques. The science of behaviour change has grown massively in the past decade but the learnings from this have not sufficiently filtered through to healthcare training and clinical practice. The reasons for this include the narrow biomedical focus in HCP education, a lack of any reinforcement value for HCPs in aiming for ‘adequate adherence’, and inadequate skills in behavioural scientists in collaborating with HCPs.

The final paper by Kardas et al. also involves the involvement of international experts to identify the key achievements of adherence research since the WHO report as well as looking ahead to the future. In addition to the more effective harnessing of new technologies, they emphasize the crucial need for a much greater recognition and prioritization of the adherence challenge at a healthcare system and policy level. Increasing clinician awareness and skill through undergraduate and postgraduate HCP training will also need to be a key element of future progress. In an era of evidence-based medicine, it is truly perplexing that the adherence issue has not been taken more seriously by health policymakers or healthcare providers (HCPs). Even though such influential organisations as the WHO and OECD have emphasized the global extent and impact of poor adherence, there is very little evidence that the situation has improved significantly in daily healthcare practice. Many years of behavioural science research has provided us with detailed evidence and insights into the nature, reasons for and impact of low adherence, not only to medication but also to other key health advice such as dietary and exercise recommendations. Quantitative and qualitative research involving people with the full spectrum of major health problems has shown that there are a wide range of cognitive, motivational and contextual reasons why people do not follow medical treatment or advice at each phase of adherence from initiation to longer term persistence.

Where does this leave us in making progress with the adherence challenge? It is obvious that there is an urgent need for all those involved in healthcare policy, training and practice to take this challenge much more seriously. The human and financial costs of non-adherence cannot be ignored any longer, and so we hope that this selection of papers will provide an impetus towards a better future.
